# How AI-based techniques for brain tumour diagnosis can address data variability

**DOI:** 10.3389/fbinf.2026.1916468

**Published:** 2026-10-05

**Authors:** Paola Lecca, Michela Lecca

**Affiliations:** 1 Faculty of Engineering, Free University of Bozen-Bolzano, Bolzano-Bozen, Italy; 2 Technologies of Vision, Fondazione Bruno Kessler, Trento, Italy

**Keywords:** data integration, data standardization, deep learning, explainable AI, glioma, medical imaging, multimodal model fusion, radiomics

## Abstract

Data variability in medical imaging of tumors refers to the inconsistencies in Magnetic Resonance Imaging and Computational Tomography scans caused by heterogeneous tumor biology, varying scanner hardware, and subjective human interpretation. This variability creates significant challenges for accurate diagnoses, treatment tracking, and AI model development. Although the variability in medical imaging data for diagnostic and prognostic purposes is a challenge for AI methods applied to other types of tumors as well, it is particularly problematic when the analysis involves gliomas and glioblastomas. Most gliomas are infiltrative and glioblastomas are highly infiltrative tumors with poorly defined margins. They exhibit significant regional variations, including cystic necrosis, localized hemorrhages, and complex microvascular proliferation. Addressing this variability requires a combination of algorithmic innovation, standardized datasets, and a leading-edge computational pipeline including traditional and AI-based data modelling methodologies to ensure diagnostic and prognostic models are robust. In this article, we will identify some AI methods that show promise in the analysis of brain tumor images and that must overcome the challenges posed by data availability. We will also offer some insights into how these challenges can be overcome and propose a possible computational pipeline that utilizes them.

## 1 Introduction

Gliomas are primary tumors of the central nervous system (brain or spinal cord) that originate from glial cells, making up approximately 40% of brain tumors. Brain gliomas are infiltrative and heterogeneous tumors that pose difficulties in early detection and effective treatment management. Artificial Intelligence (AI) has the ability to transform the field of neuro-oncology and can improve glioma detection, imaging segmentation, and non-invasive molecular characterization more effectively than traditional diagnostic methods through deep learning-based radiomics and radiogenomics (Menze et al., 2021; Voigtlaender et al., 2025). However, data variability serves as a significant challenge to the application of AI in neuro-oncology for the treatment of gliomas. Although AI models demonstrate impressive accuracy in controlled experiment - especially regarding tumor segmentation and classification - variability in data sources, imaging protocols, and patient demographics greatly hinders their generalization and clinical use. Furthermore, substantial variability in molecular, histological, and radiological characteristics, results in unpredictable tumor behavior, treatment reactions, and patient results. This drastic variability is seen both among different patients (inter-tumoral) and within the same patient (intra-tumoral), resulting in a recurrence rate exceeding 90% for glioblastomas (see (Ivy Center, 2026; van der Sanden et al., 2016; Jørgensen et al., 2023; Patricoski-Chavez et al., 2025; Sleptsova et al., 2026) for detailed data on recurrence percentage of gliomas and glioblastomas).

Gliomas range from lower-grade tumors to aggressive glioblastomas, they vary massively in their form, cellular makeup, and location, making standard models difficult to generalize (Reddy et al., 2025). Furthermore, a single glioma can contain varying grades of malignancy, necrotic cores, and surrounding edema (Hu et al., 2020), and tumor growth distorts surrounding brain structures and ventricles, warping the spatial landmarks that automated algorithms rely on. Five ways data variability impairs AI-based glioma diagnosis include the following. (i) Imaging variability: differences in scanners, field strengths, sequences, and preprocessing across institutions cause AI models to perform poorly on new datasets. (ii) Limited datasets: reliance on small, single-center data fails to capture patient diversity, leading to model overfitting. (iii) Temporal contamination: older data lack modern molecular profiling, tainting datasets with obsolete information. (iv) Tumor heterogeneity: high biological, cellular, and genetic variation within and between gliomas makes tumor behavior difficult to predict reliably. (v) Radiomics challenges: high-dimensional imaging features are highly sensitive to variations in segmentation strategies, spatial dimensions, and distinguishing edema from true tumor infiltration. In Supplementary Material, Section 1 explores these aspects in greater depth. In turn, data variability affects the complexity of a deep neural network by increasing the required model capacity, training time, and risk of overfitting, finally contributing to the opaqueness of AI algorithms (more details in Section 2 of Supplementary Material).

First, in this perspective article, based on an in-depth analysis of recent literature on the subject, we wish to highlight the methodologies that can reduce the variability of the input data for the most advanced AI algorithms, whilst also explaining the mechanisms through which data variability can undermine the promises of AI, especially deep neural networks. Secondly, building on the understanding of these mechanisms, we provide a computational pipeline. This pipeline embodies a conceptual framework designed precisely for the translation of raw data characterised by high variability and heterogeneity into predictive models the diagnosis and prognosis of brain tumors.

## 2 Data variability and AI-based approaches facing its challenges

Addressing data variability in gliomas with AI necessitates a comprehensive approach that considers both the natural diversity of the tumor and the differing technical aspects of imaging and molecular data among various institutions (AI models often fail to generalize across hospitals due to protocol-specific biases). Essential strategies encompass the following actions.

- *Applying uniform data preprocessing and annotation standards.* Variability in imaging techniques (scanners, magnetic field strengths, reconstruction algorithms) leads to *batch effects*, i.e., non-biological, technical differences in medical images. These effects can lead to incorrect, biased results when training artificial intelligence models or analyzing multi-site data. Batch effects are particularly problematic for radiomics and deep learning, as they introduce variations in intensity, contrast, and texture that are not related to the patient’s biology (Hu et al., 2023; Stamoulou et al., 2022). Practical implementation of data preprocessing to minimize data variability consists of (i) normalizing data through strict pre-processing methods, such as logarithmic transformation, quantile normalization, and z-score scaling, (ii) employing tools to unify multi-platform datasets (for instance, ComBat, Harmony, or Limma (Tran et al., 2020; Chazarra-Gil et al., 2021; Lee et al., 2023; D’Anna et al., 2024; Patikas et al., 2026)), and (iii) implementing coordinated annotation protocols such as tackling noise in expert assessments by developing standardized, and multi-observer agreement directives for tumor segmentation to minimize human-generated bias.
- *Leveraging AI frameworks such as federated learning (FL)* (Li et al., 2026b; Saeed et al., 2025; Moshawrab et al., 2023) *and generative models to improve model generalizability.* FL is specifically designed to address data variability, diversity, and heterogeneity problems in machine learning, particularly when data is scattered across multiple, decentralized locations. While traditional machine learning requires collecting data in one place - which often fails due to privacy constraints or data silos - federated learning is a decentralized machine learning approach where AI models are trained across multiple devices or edge servers (like smartphones or IoT devices) without sharing raw data. Instead of centralizing data, devices train a local model and send only parameter updates to a central server to improve a shared global model, enhancing privacy and reducing communication overhead. FL would enable AI models to learn from decentralized data across various institutions without exchanging raw patient information, maintaining privacy while benefiting from diverse, varied datasets.
- *Integrating radiomics features from MRI with molecular, histological, and clinical data to create a comprehensive biological landscape of the tumor.* Using advanced AI techniques (e.g., deep learning feature fusion (Zhang et al., 2024)) to combine imaging features with omics data (genomics, transcriptomics) improves prediction of survival and treatment response.
- *Using AI techniques to explore peritumoral areas.* These zones in glioblastomas are not merely uniform edema zones; they contain infiltrating tumour cells and varied immune responses often overlooked by human specialists (Zhang et al., 2023; Elmahdy and Sebro, 2023; Azemi and Di Ieva, 2025; Liu H. et al., 2025; Linton-Reid et al., 2026). Variability in data within the peritumoral region is a primary concern in advanced oncology and medical imaging. Since this area serves as the battleground between the tumor and the host’s immune system, changes in its biological and imaging characteristics offer essential information regarding disease severity and treatment effectiveness (Xu et al., 2024; Liu Y. et al., 2025; Zhao Q. et al., 2025). The peritumoral region is irregular. It shows organized spatial gradients of biological activity that vary with increasing distance from the tumor edge (Triantafyllou et al., 2026). in Table 1, we identify four core AI techniques that show promise for the analysis of brain tumours, but are particularly hampered by data variability and by the characteristics of the peri-tumoural area, which is so important for prognostic purposes.
- Explainable AI (XAI) glioma detection mitigates data variability in medical imaging by contributing transparency in deep learning decisions (Muhammad and Bendechache, 2024; Marchi, 2026). XAI hold aims to transform unclear predictions into understandable visual information, fostering clinical trust, facilitating subgroup-specific audits to reveal concealed algorithmic bias, and averting misdiagnoses that may occur when AI models aim to generalize across diverse clinical environments (Ray, 2026). In other words, XAI helps us understand how the variability of data processed using AI techniques affects the results in two ways: by extreme context-specificity and undesirable generalization. See Supplementary Material, Section 3 for further details.

**TABLE 1:** Data variability in neuro-oncology significantly hampers AI techniques. Here we list the causes of data variability that have the greatest impact on AI performance in the various areas of neuro-oncology in which it is applied.

AI techniques	Causes of data variability
AI-assisted radiomics	Radiomics examination of the imaging area adjacent to a tumor can benefit from the usage of earning to identify microstructural, vascular, and immunological alterations in the tissue stroma or edema, greatly improving the precision of cancer staging, prognosis, and treatment response forecasts (Dou et al., 2018; Li et al., 2026a). Due to traditional imaging primarily emphasizing active, contrast-positive centers, the non-enhancing, invasive borders tend to be inadequately depicted. Nonetheless, the recent literature reports about *virtual biopsy*, a combination of sophisticated radiomics and machine learning able to categorize molecular subtypes without the need for invasive biopsies (Holtkamp et al., 2025; Liu et al., 2021; Murray et al., 2022)
Convolutional neural networks (CNNs)	CNNs are utilized to examine the spatial and temporal variability of the peritumoral area, frequently surpassing conventional techniques. CNNs can effortlessly detect hierarchical, pixel-level characteristics in 3D and 4D medical imaging at multiple scales, identifying subtle microstructural and perfusion variations that manual radiomics and traditional texture analysis may miss (Wang et al., 2024; Li et al., 2026a)
Multimodal fusion models (MFMs)	These models combine various imaging information to illustrate tumor infiltration, particularly in gliomas. They integrate the detailed anatomy provided by standard MRI (T1, T2, FLAIR) with functional, microstructural, and metabolic information from advanced methods (ADC, DTI, perfusion) to define tumor borders beyond typical contrast enhancement. CNNs serve as the core of MFMs, capturing detailed local features from medical images to enhance the overall context offered, for instance, by multi-omics data (Alleman et al., 2023; Yi et al., 2025; Saueressig et al., 2026). MFMs are promising tools (Liang et al., 2025), but they are not without challenges that still need to be overcome. Specifically, we identify two major challenges as follows. Multimodal architectures often require multi-branch deep learning networks with independent backbones (e.g., CNNs for imaging). Scaling these simultaneously dramatically increases parameter counts, tensor sizes, and memory footprints, making real-time processing or rapid training on standard hardware difficult without significant optimization (Al-Zoghby et al., 2025). Furthermore, harmonizing different modalities (such as clinical text, genomic sequences, and high-resolution medical images) is non-trivial. Modalities exhibit heterogeneous structures, variable sampling rates, and different semantic resolutions. Bridging this gap requires advanced alignment methods (e.g., cross-modal attention (Brindha and Kavitha, 2026; Driver and Spence, 1998)) to weight data properly without losing modality-specific characteristics
Machine learning classifiers	Algorithms such as support vector machines (SVM) (Wang, 2022), Random forests (Breiman, 2001), XGBoost (Chen and Guestrin, 2016), and CatBoost (Prokhorenkova et al., 2018) are commonly used to train on these features to predict recurrence. Through the examination of the broad array of features derived from radiomics, machine-learning methods can improve tumor diagnosis, evaluate treatment response, and forecast patient outcomes. More specifically, ML classifiers examine the peritumoral region of gliomas and glioblastomas to distinguish between pure vasogenic edema and infiltrative tumor cells, forecast recurrence, and non-invasively map molecular signatures (Tabassum et al., 2023; Michalska-Foryszewska et al., 2024)

### 2.1 Data variability as a reason of lack of feature reproducibility

The variability of radiomic data also affects the reproducibility of radiomic features. Unstandardized image preprocessing (e.g., interpolation, intensity discretization) and differing computational algorithms frequently lead to discordant feature values on identical datasets. Reproducibility can be established in two stages: imaging reproducibility (i.e., test-retest stability of a scan) and statistical reproducibility (i.e., robustness of features to segmentation and feature extraction software) (Ibrahim et al., 2021). Feature reduction and compliance to Image Biomarker Standardization Initiative (IBSI) are proposed to ensure mathematical harmonization, software interoperability, and cross-institutional reproducibility of MRI-derived tumor phenotypes, but they are not without their drawbacks, as highlighted in Supplementary Table S1 in Supplementary Material.

It should also be noted that reproducibility and standardization are not independent of transparency, as discussed in Supplementary Material, Section 4).

## 3 An operative pipeline to mitigate the effects of data variability

The strategies we have listed in points a) – e) in the previous section as possible actions to be taken to ensure that the impact of data variability on AI methodologies is mitigated and that, at the same time, AI can manage this variability without compromising its modelling and predictive power, do not, when used in isolation, solve the problem of data variability, but merely address specific, context-dependent aspects of it. As a way forward, we propose a possible framework in which each of the listed strategies works in concert with the others, as illustrated in Figure 1. The framework is organised into three levels: the first level, in which federated learning and data pre-processing procedures operate in parallel to standardise the data. There is then a second level that processes the outputs obtained in the first layer and involves data integration which integrates imaging characteristics with clinical data, genomics, and multi-omics to enhance precision medicine, surpassing single-source models in forecasting disease advancement and treatment responses (Farina et al., 2023; Marouf et al., 2025). AI techniques (radiomics analysis, CNNs, Multimodal fusion models and machine learning classifiers), situated at the third level of the data processing and modelling framework, are applied to the integrated data. We anticipate that, in the immediate and near future, as far as glioma and glioblastoma are concerned, these techniques will need to be tailored to the peri-tumoural area not only because this zone is a primary site of microscopic tumor cell invasion and the source of the majority of recurrences following surgery (Trevisi and Mangiola, 2023; Ballestín et al., 2024), but also because its spatial heterogeneity is not well studied (Wang et al., 2024), and AI techniques should be used, tested and empowered to explore, identify and prognostically classify these heterogeneities. At the third level we also place the XAI *post hoc* explainers, which employ methods such as feature attribution models (e.g., SHAP, lime) to emphasize crucial microenvironment features on the brain image to aid radiologists in the diagnosis. XAI clarifies imaging in three ways (Lu et al., 2025): (i) by highlighting which texture or shape metrics drive a risk score: (ii) by pinpointing exact zones in the tissue surrounding a tumor influencing an stereotactic body radiotherapy outcome prediction, and (iii) by replacing hidden computations with clear visual evidence matching medical knowledge. By pinpointing the specific quantitative characteristics in the tissue around a tumor that influence an AI’s decision, doctors can validate and trust non-invasive biomarkers for early diagnosis and treatment strategies. Post-hoc explainers can be utilized, as they offer numerical values indicating the precise contribution of a particular peritumoral texture to the model’s prediction, thus enhancing the transparency and clinical reliability of the AI’s reasoning.

**FIGURE 1:**
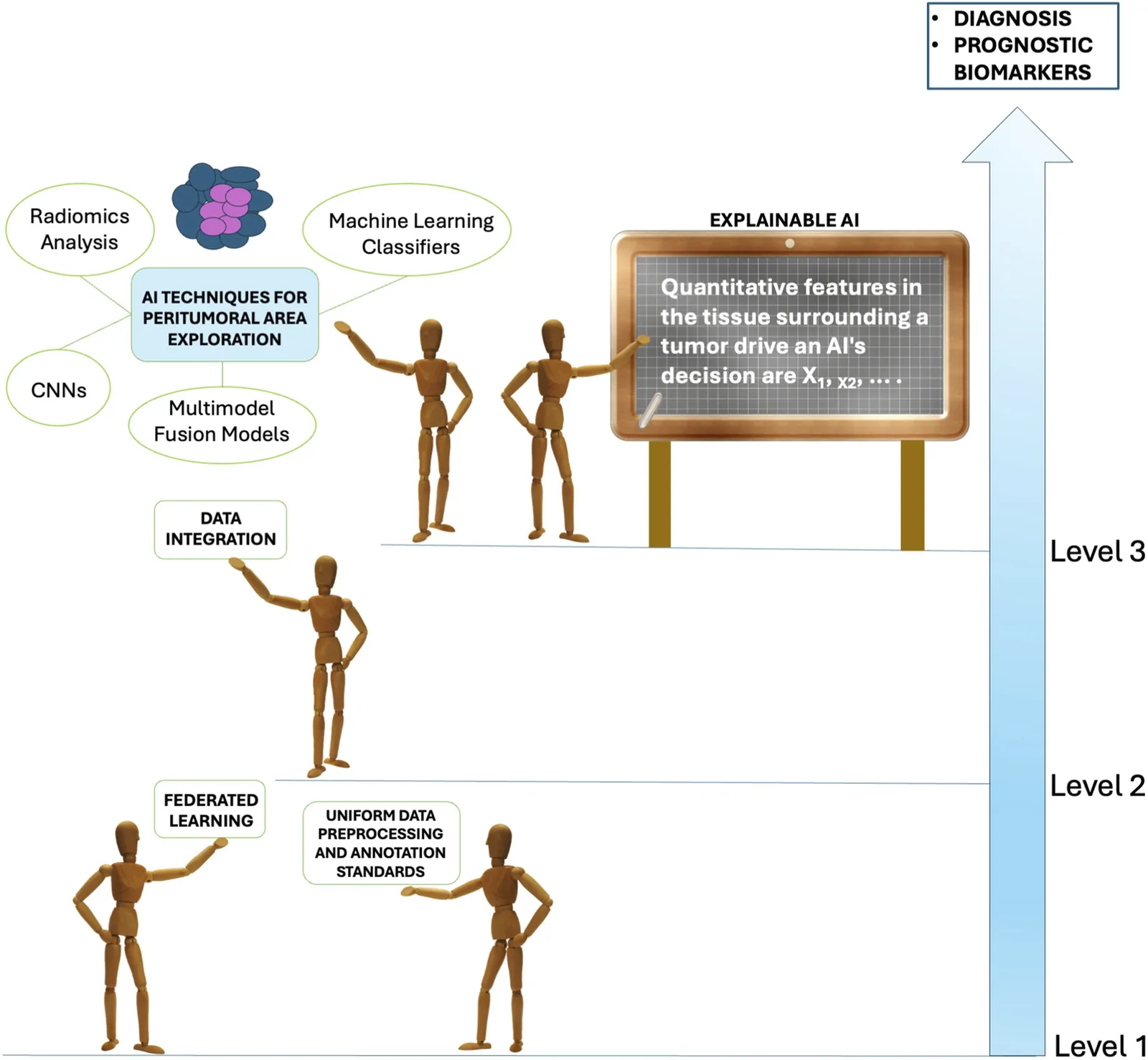
Illustrative diagram of a framework for the preprocessing of biological and medical data and for their processing with artificial intelligence techniques specifically designed to support the physician in the evaluation of brain tumor lesions such as gliomas and glioblastomas. Starting with clinical and biological data as input, and using methodologies and objectives that we classify into three levels (federated learning and data standardisation (Level 1), data integration (Level 2), and finally the application of AI techniques (Level 3)), the computational framework produces an output consisting of the generation of information and the extraction of features to support diagnosis and the identification of prognostic biomarkers. The pathway from Level 1 to Level 3 is not without its obstacles or challenges. Specifically, the data integration process is a particularly delicate and important stage, given that the integration of MRI, and PET scans with genomic and clinical profiles transforms glioma diagnostic analyses into a non-invasive, data-driven process. The greatest challenge that the data integration process is likely to face in the near future is the choice of a suitable integration method that mitigates overfitting (if not prevent) – a risk that is becoming increasingly likely due to the continuous refinement of data integration techniques and the ever-increasing volume of data to be integrated. Explainable AI plays also an important role in the pipeline, as it tackles the “black box ”characteristic of deep learning in neuro-oncology by elucidating which MRI or genetic attributes influence AI models. XAI applied to the peritumoral brain are can help clinicians differentiate between tumor infiltration and vasogenic edema on non-invasive advanced neuroimaging.

Level 3 is central to the pipeline shown in Figure 1, as it effectively serves as the foundation for the implementation of non-invasive analyses for diagnostic and prognostic purposes. Choosing an appropriate data fusion method is strategic to forecast particular molecular subtypes (e.g., wild-type versus mutant) and identify microscopic infiltration areas prior to surgical biopsy. The data fusion methods commonly used are known as early fusion, late fusions and intermediate fusion. Early fusion which combines radiomic and genomic data prior to entering them into a diagnostic model. This enables profound cross-modal interactions, and that is a benefit since knowing these interactions means knowing how the data and models influence, inform, and combine with each other, but it presents an increased chance of overfitting (Zhao L. et al., 2025; Marouf et al., 2025). Late fusion treats each data type (e.g., MRI scans vs. genomics) using separate, independent workflows, merging their results solely at the final decision-making stage. Since the models do not interact during the training process, late fusion may miss intricate, cross-modal interactions or cooperative biomarkers that develop in the earlier phases of representation. To address this, researchers frequently investigate intermediate fusion or hybrid architectures that link networks prior to the final decision. Hybrid/intermediate fusion techniques utilize sophisticated deep learning frameworks, including Transformer-3D CNN hybrid models featuring cross-attention. These networks focus on the most crucial genomic markers, such as isocitrate dehydrogenase (IDH), while also examining spatial MRI/PET characteristics. IDH mutations are indeed critical diagnostic and prognostic genomic markers primarily used in the classification of diffuse gliomas (Solomou et al., 2023).

## 4 Discussion

At the moment of writing, computational pipelines that integrate data variability mitigation methods at different levels of a computational global, strongly data-integrative pipeline and address various aspects of the raw data to be processed are largely absent from the literature. In literature we can find, however, computational pipelines that specifically outline the steps required to apply each of the single approaches we organised into the three levels of Figure 1. Unfortunately, in the majority of cases, these computational frameworks have limited clinical application due to the fact that standardizing biological and imaging metrics is complex. The reason lies in the fact that standardising data is a complex process, and for this very reason, fragmenting this standardisation by confining it to specific purposes and methods not only contributes to increasing the *uncontrollability* of data variability (Taha et al., 2021), but it also represents a considerable effort that does not pay off in terms of achieving the desired outcome, namely, a diagnosis that is interpretable, verifiable and accurate.

The pipeline we propose is a hypothetical one, a vision, in fact, but compared to current proposals, which are context- and method-specific and yet often remain purely conceptual, it aims to address the problem of mitigating data variability in neuro-oncology on a global scale. It therefore presents itself as a challenge that may be worth tackling, given that the benefits in terms of supporting neuro-oncological diagnosis could be significant. On the contrary, the pipeline we propose in Figure 1 aims to address the problem of mitigating data variability in neuro-oncology on a global scale. It therefore presents itself as a challenge that may be worth tackling, given that the benefits in terms of supporting neuro-oncological diagnosis could be significant. At the present, the state-of-art conceived strategy to approach the issue of data variability is a pipeline-based fusion that sequentially integrates image-level harmonization, robust deep learning architectures, and statistical correction techniques. This multi-stage pipeline provides strong robustness, but combining these methods introduces distinct trade-offs across four main areas, e.g., (i) data distortion and artifact generation (especially if generative AI methods are used), (ii) high computational and data demands, (iii) pipeline complexity and cascading errors, (iv) destruction of biological meaningful variance. See for instance (Ouhmouk et al., 2025; Ho et al., 2026; Khalighi et al., 2024; Bakas et al., 2024; Taha et al., 2021; Kamga et al., 2025; Li and Huang, 2020), as example of literature references highlighting the issues (i) - (iv). Problems (i) and (iv) are particularly undesirable for the following reasons. Generative AI (such as diffusion models and Generative Adversarial Networks (Yazdani et al., 2025)) can introduce artificial, non-existent features (hallucinations or artifacts) into synthetic medical data. While used to augment MRI scans or predict tumor growth, generated outputs carry the risk of presenting false anatomical structures that could lead to misdiagnosis or flawed surgical planning (Xia et al., 2025; Parchur et al., 2025). Concerning the statistical corrections, once the statistical correction factors [like ComBat parameters (Reynolds et al., 2023)] are locked post-training, they cannot dynamically adapt to real-time, single-patient clinical inference without recalculating cohort statistics. Since our pipeline does not involve the use of generative AI nor the implementation of statistical corrections, it aims to reduce, if not eliminate, the risks associated with trade-offs (i) and (iv). Regarding the elimination of statistical adjustments to avoid the risk of eliminating biological relevant variance (trade-off (iv)), we clarify that, indeed, statistical corrections in AI can inadvertently eliminate or obscure biologically relevant variance. While harmonization techniques are vital for mitigating technical artifacts Bakas et al. (2024); Dommalapati et al. (2025); Voigtlaender et al. (2025); Hu et al. (2023); Beer et al. (2020); Nan et al. (2023); Reynolds et al. (2023), over-correction poses a significant risk to biomarker discovery and personalized medicine (Hui et al., 2024).

In conclusion, mitigating the variability of the input data for these methods can therefore be beneficial when analysing such a heterogeneous area. Whilst research into AI methods in radiomics (Level 3 in the pipeline) is making great strides, it is becoming crucial to focus the research more on two delicate stages: data integration and the use of XAI for data processing in peritumoural area. Both data integration and XAI act as bridges between humans and machines, which must be robust and reliable to avoid falling into medical errors with unfortunate outcomes.

## Data Availability

The original contributions presented in the study are included in the article/Supplementary Material, further inquiries can be directed to the corresponding author.
