# Supplementary material for: How AI-based techniques for brain tumour diagnosis can address data variability
Source: Front Bioinform. 2026 Oct 5;6:1916468. doi: 10.3389/fbinf.2026.1916468 (PMC13677829; doi:10.3389/fbinf.2026.1916468)
Supplement: Supplementary file 1 [file DataSheet1.pdf]

Supplementary Material: How AI-based techniques for brain tumour diagnosis can address data variability

Paola Lecca [1], Michela Lecca [2]
[1]Faculty of Engineering, Free University of Bozen-Bolzano, Bolzano , Italy
[2] Technologies of Vision, Fondazione Bruno Kessler, Trento, Italy

1 HOW DATA VARIABILITY IMPAIRS AI ALGORITHMS

Consultation of recent literature (e.g. (Rahimi and Rahimi, 2023; Björklund et al., 2023; Hassija et al., 2023; Fares et al., 2024; Fan et al., 2024a; Peracchio et al., 2024; Awuah et al., 2025; Voigtlaender et al., 2025)) has allowed us to identify five ways data variability currently impairs the use of AI-based techniques for the diagnosis of gliomas. We will briefly describe them in the following.

- Imaging variability: Magnetic Resonance Imaging (MRI) data frequently differs between institutions because of variations in scanners, magnetic field intensities, imaging sequences, and preprocessing methods. This variation in image look, contrast, and quality leads AI models trained on one dataset to perform poorly when used with another, hindering the ability of the algorithm to perform effectively when applied to new domains, datasets, or tasks different from its original training environment.
- Limited, non-representative datasets: Deep learning models need large quantities of data, which are frequently unavailable in neuro-oncology. Dependence on limited or single-center datasets does not accurately reflect the genuine diversity of patient populations and the complexity of tumor biology, resulting in overfitting and subpar performance in actual situations.
- Contamination from temporal data changes: A large portion of data for glioma classification originates from previous periods that lacked modern molecular profiling, tainting datasets with obsolete information.
- Intra- and inter-tumoral heterogeneity: Gliomas, particularly high-grade gliomas, show considerable biological, cellular, and genetic variation. This inherent variability is hard to measure and standardize, making it challenging for AI to reliably forecast tumor behavior. Finally,
- (v) Radiomics challenges: The suffix “-omics ”indicates complex high-dimensional data extracted from few samples (Gillies et al., 2016). In this context, radiomics indicates MRI, CT or PET images coupled with a set of quantitative features describing size, shape, volume, texture, intensity or gradient distributions of a region of interest (ROI), i.e. a brain part possibly affected by glioma. Methodological variability in ROIs definition is another significant source of data variability that stems from the difficulty in distinguishing vasogenic edema from actual tumor infiltration, differing segmentation strategies, spatial dimensions, and the tumor heterogeneity. This variability remarkably impacts the feature extraction, which currently is often made by machine/deep learning techniques (Lao et al., 2017; Tabatabaei et al., 2021; Fan et al., 2024b).

2 EXPLAINABLE AI AND DATA VARIABILITY

Data variability is closely related to the opaqueness of AI algorithms (often referred ad “black box”), which means that clinicians to be reluctant to trust AI-generated predictions about tumour grade, molecular

markers, or survival forecasts. Specifically, data variability due to the range, noise, and statistical fluctuations in training data, is inherently linked to the opaque structure of AI algorithms, as it affects the complexity required for the model to perform optimally. Significant data variability compels models to evolve into more complex and less transparent forms, as they need to comprehend non-linear relationships and complicated, high-dimensional patterns for precision, leading to a black box model where understanding the decision-making process is challenging. In fact, at the architectural level, to manage high data variability, deep neural network models employ numerous interconnected layers. These layers learn nonlinear representations of input features, which creates high predictive accuracy but obscures the reasoning behind specific outputs. Precisely, deep neural networks, which stack multiple layers, act as hierarchical feature extractors capable of mapping highly non-linear relationships, whereas high interconnection allows the network to learn higher-order statistics and complex correlations. Complex models often exhibit extreme sensitivity to small changes in input data, known as *adversarial vulnerability*. The most common consequence of high data variability and the correspondent significant structural complexity of the neural network is the *overfitting*, of which the high sensitivity to inputs is a sign. In overfitting, the model fails to distinguish between the meaningful underlying signal and random noise and "memorizes "the specific training set rather than learning patterns that generalize to new data.

3 EXPLAINABLE AI IN NEURO-ONCOLOGY: BETWEEN MATHEMATICS AND CLINICAL INTUITION

The bulky structure of artificial neural networks makes hard to understand and validate the algorithm outcomes, especially for AI non expert users. XAI attempts to disentangle the network response by highlighting image regions or pixels and features that contribute most to the diagnosis and thus providing a visual explanation of the result, that are of primarily importance both for the prediction assessment in charge of medical doctors and the patients' right to explanation. Examples of well known XAI tools, typically combined together for supporting and/or integrating traditional diagnosis approaches (e.g. (Rahman et al., 2024, 2025; Nohara et al., 2022; Gundogan, 2025; Osadebey et al., 2023; Zeineldin et al., 2022; Christodoulou et al., 2026b)) are Grad-CAM (Gradient-weighted Class Activation Mapping) (Selvaraju et al., 2020), Local Interpretable Model-agnostic Explanations (LIME) (Zafar and Khan, 2021), Shapley Additive exPlanations (SHAP) (Nohara et al., 2022). LIME and SHAP are algorithmic tools used in XAI to assign importance scores to input variables (see (Nuraini et al., 2025) for a report on their performance). Christodoulou et al. (Christodoulou et al., 2026a) study proves to be also of interest in the context of XAI. The authors present ASLNet, an interpretable 3D residual network architecture trained on arterial spin labelling (ASL) MRI to forecast histopathologic grade and overall survival in individuals with diffuse glioma. These approaches enables a better understanding of the AI prediction, but they still depend on the training set and data quality, making still necessary the supervision of medical staff for a reliable translation of the AI response into a clinical diagnosis.

It should be also noted that XAI does not guarantee clinical validity (i.e. the accuracy with which a medical test predicts the presence, absence, or risk of a specific clinical condition or outcome). However, XAI aims at bridging the gap between complex mathematical models and clinical intuition by converting opaque ("black box") decisions into transparent, verifiable insights. XAI doesn't completely crack open a neural network to reveal its exact step-by-step reasoning, but it translates opaque model behaviors into human-understandable terms. Rather than fully unraveling the "black box", XAI acts as a translator, shedding light on why an AI made a specific decision. Furthermore, XAI highlights key parts of data, for instance specific pixels in an image that the model focused on, and implement the feature importance

ranking. This XAI action is of crucial importance, since we cannot carry out any experimental checks unless we are aware of the steps and line of reasoning that led to a diagnosis or the identification of a cause. In other terms, without knowing this reasoning, we cannot carry out any experiments to confirm or refute the hypotheses put forward. Furthermore, without the knowledge of the reasoning line, at best, we can rely on statistical data or associative reasoning, which in clinical practice are very weak tools for identifying a cause, its mechanisms of action and designing the therapeutic measures to be taken, particularly in precision and personalised medicine. This is often referred to as "the trap of associative reasoning "(Ferreira-da Silva et al., 2025; Kabir et al., 2025; Bhinder et al., 2021).

There is no doubt that XAI is crucial in radiomics. Due to these reason more efforts need to be done to overcome three critical limitations of XAI under dataset shift and multicenter variability: (i) feature misalignment, where explanations highlight scanner artifacts rather than true biological markers; (ii) explanation unreliability, where methods like SHAP (Nohara et al., 2022) or Grad-CAM (Selvaraju et al., 2020) yield fragmented or anatomically misaligned heatmaps; and (iii) generalization gaps across diverse clinical protocols.

4 FEATURE REPRODUCIBILITY, STANDARDIZATION AND TRANSPARENCY

Reproducibility and standardization are not independent of transparency; on the contrary, transparency is an essential prerequisite. Currently, we dispose of many neuro-oncology AI models that promise to be predictive, but fail to explain their data processing pipelines. Because brain anatomy is highly complex, hidden biases (like including normal brain tissue in the tumor bounding box) often go unnoticed. In this context TRIPOD-AI and CLAIM reporting guidelines support transparency in neuro-oncological research aided by AI (Collins et al., 2024; Klontzas et al., 2023). They mandate the disclosure of how skull-stripping was performed, how brain atlas co-registration was handled, and exactly how missing MRI sequences (e.g., a missing Fluid-Attenuated Inversion Recovery) or T2 scan (Dhabalia et al., 2024) were managed.

5 SUPPLEMENTARY TABLE

Solutions to feature reproducibility	Comments and perspetives
Feature reduction	Feature reduction might act as a partial solution to the problem of feature of reproducibility. In fact feature reduction (e.g., using Intraclass Correlation Coefficients, or ICCs (Li et al., 2025)) could be used to retain only stable, non-redundant features prior to model training (Lee et al., 2024). Brain tumors contain mixed regions of active contrast-enhancing tumor, non-enhancing tumor, central necrosis, and surrounding vasogenic edema. Manually outlining these complex volumes of interest introduces massive inter-observer variability among neuroradiologists. Evaluating feature reproducibility via ICCs is critical here. By having multiple clinicians segment the same brain tumor, researchers can mathematically filter out features that fluctuate based on human drawing errors, keeping only the most robust features for model building.
Compliance to IBSI (Image Biomarker Standardization Initiative)	IBSI provides an independent international collaboration to standardize the extraction of quantitative image biomarkers. This collaboration establishes rigorous mathematical definitions, nomenclature, and uniform image processing workflows (spanning acquisition, interpolation, discretization, and feature aggregation). By adopting IBSI-compliant platforms (such as PyRadiomics (van Griethuysen et al., 2017) or LIFEx (Nioche et al., 2018)), researchers can ensure cross-software agreement, avoid mathematical ambiguities, and improve multicenter workflow compatibility, that are prerequisites necessary to enable XAI to overcome its current limitations, as already mentioned in point e) of the previous Section.
Graph-Based Feature Selection (Graph-FS)	Instead of treating radiomic metrics as isolated variables, Graph-FS transforms the entire feature pool into a network map. Every extracted radiomic feature is mapped as a single node in a graph. Connections (edges) between nodes are weighted using pairwise statistical dependencies, such as mutual information coefficients, Pearson correlations, or partial correlations (e.g., Graphical Lasso (Mazumder and Hastie, 2012)). Then, the algorithm searches for highly dense subgraphs or connected components. Features clustered tightly together represent highly redundant variations of the same underlying tumor biology. The advantage of Graph-FS methods is that instead of randomly picking or forcefully keeping all correlated variables, the algorithm extracts the central, most topologically stable vertex from each cluster to act as the signature representative. GRAPH-FS is expected to prove to be a particularly effective method. Indeed, recent multi-center oncology validation studies demonstrate a massive disparity in robustness between graph-based frameworks and classic machine learning selectors Moradmand et al. (2025).
Cross-center validation	Data variability in radiomics is responsible of the overfitting on center-specific features. Consider for instance the following example. It is known that machine learning models are incredibly sensitive. Instead of learning the biological markers of glioma recurrence, a model trained on a single institution's data might inadvertently learn the specific image noise or reconstruction kernel unique to that hospital's MRI scanner. External validation across institutions is then the ultimate stress test for this issue. If a neuro-oncology model achieves a high area under the curve (AUC $= 0.90$) at Center A, but drops to AUC $= 0.55$ when tested on an external dataset from Center B, it indicates the model overfits to Center A's specific scanner settings rather than the biological characteristics of the brain tumor.

Table S1. Here we summarize some methods whose application helps ensure the reproducibility of features, highlighting obstacles that still need to be overcome and indicating which method, based on current knowledge, appears to be more promising than others, e.g. Graph-FS.

REFERENCES

Awuah, W. A., Ben-Jaafar, A., Roy, S., Nkrumah-Boateng, P. A., Tan, J. K., Abdul-Rahman, T., et al. (2025). Predicting survival in malignant glioma using artificial intelligence. *European Journal of Medical Research* 30. doi:10.1186/s40001-025-02339-3

Bhinder, B., Gilvary, C., Madhukar, N. S., and Elemento, O. (2021). Artificial intelligence in cancer research and precision medicine. *Cancer Discovery* 11, 900–915. doi:10.1158/2159-8290.cd-21-0090

Björklund, A., Henelius, A., Oikarinen, E., Kallonen, K., and Puolamäki, K. (2023). Explaining any black box model using real data. *Frontiers in Computer Science* 5. doi:10.3389/fcomp.2023.1143904

Christodoulou, R. C., Vamvouras, G., Papageorgiou, P. S., Vassiliou, E., Papageorgiou, S. G., Kalogeropoulou, C., et al. (2026a). Aslnet: an explainable deep learning framework for glioma grading and survival prediction. *Frontiers in Oncology* 16. doi:10.3389/fonc.2026.1818663

Christodoulou, R. C., Vamvouras, G., Pitsillos, R., Solomou, E. E., and Georgiou, M. F. (2026b). Explainable, modality-adaptive radiomics for mgmt methylation prediction in high-grade glioma: a decision-curve analysis study. *Frontiers in Oncology* 15. doi:10.3389/fonc.2025.1731258

Collins, G. S., Moons, K. G. M., Dhiman, P., Riley, R. D., Beam, A. L., Van Calster, B., et al. (2024). Tripod+ai statement: updated guidance for reporting clinical prediction models that use regression or machine learning methods. *BMJ* , e078378doi:10.1136/bmj-2023-078378

Dhabalia, R., Kashikar, S. V., Parihar, P. S., and Mishra, G. V. (2024). Unveiling the intricacies: A comprehensive review of magnetic resonance imaging (mri) assessment of t2-weighted hyperintensities in the neuroimaging landscape. *Cureus* doi:10.7759/cureus.54808

Fan, H., Luo, Y., Gu, F., Tian, B., Xiong, Y., Wu, G., et al. (2024a). Artificial intelligence-based mri radiomics and radiogenomics in glioma. *Cancer Imaging* 24. doi:10.1186/s40644-024-00682-y

Fan, H., Luo, Y., Gu, F., Tian, B., Xiong, Y., Wu, G., et al. (2024b). Artificial intelligence-based mri radiomics and radiogenomics in glioma. *Cancer Imaging* 24, 36

Fares, J., Wan, Y., Mayrand, R., Li, Y., Mair, R., and Price, S. J. (2024). Decoding glioblastoma heterogeneity: Neuroimaging meets machine learning. *Neurosurgery* 96, 1181–1192. doi:10.1227/neu.0000000000003260

Ferreira-da Silva, R., Cruz-Correia, R., and Ribeiro, I. (2025). Beyond black boxes: using explainable causal artificial intelligence to separate signal from noise in pharmacovigilance. *International Journal of Clinical Pharmacy* 48, 677–681. doi:10.1007/s11096-025-02004-z

Gillies, R. J., Kinahan, P. E., and Hricak, H. (2016). Radiomics: images are more than pictures, they are data. *Radiology* 278, 563–577

Gundogan, E. (2025). A novel hybrid deep learning model enhanced with explainable ai for brain tumor multi-classification from mri images. *Applied Sciences* 15, 5412

Hassija, V., Chamola, V., Mahapatra, A., Singal, A., Goel, D., Huang, K., et al. (2023). Interpreting black-box models: A review on explainable artificial intelligence. *Cognitive Computation* 16, 45–74. doi:10.1007/s12559-023-10179-8

Kabir, S., Hossain, M. S., and Andersson, K. (2025). A review of explainable artificial intelligence from the perspectives of challenges and opportunities. *Algorithms* 18, 556. doi:10.3390/a18090556

Klontzas, M. E., Gatti, A. A., Tejani, A. S., and Kahn, C. E. (2023). Ai reporting guidelines: How to select the best one for your research. *Radiology: Artificial Intelligence* 5. doi:10.1148/ryai.230055

Lao, J., Chen, Y., Li, Z.-C., Li, Q., Zhang, J., Liu, J., et al. (2017). A deep learning-based radiomics model for prediction of survival in glioblastoma multiforme. *Scientific reports* 7, 10353

Lee, S. B., Hong, Y., Cho, Y. J., Jeong, D., Lee, J., Choi, J. W., et al. (2024). Enhancing radiomics reproducibility: Deep learning-based harmonization of abdominal computed tomography (ct) images. *Bioengineering* 11, 1212. doi:10.3390/bioengineering11121212

Li, Y., Jiang, J., Li, X., and Zhang, M. (2025). Multi-modal data integration of dosiomics, radiomics, deep features, and clinical data for radiation-induced lung damage prediction in breast cancer patients. *Journal of Radiation Research and Applied Sciences* 18, 101389. doi:10.1016/j.jrras.2025.101389

Mazumder, R. and Hastie, T. (2012). The graphical lasso: New insights and alternatives. *Electronic Journal of Statistics* 6. doi:10.1214/12-ejs740

Moradmand, H., Molitoris, J., Ling, X., Schumaker, L., Allor, E., Thomas, H., et al. (2025). Graph feature selection for enhancing radiomic stability and reproducibility across multiple institutions in head and neck cancer. *Scientific Reports* 15. doi:10.1038/s41598-025-12161-w

Nioche, C., Orlhac, F., Boughdad, S., Reuzé, S., Goya-Outi, J., Robert, C., et al. (2018). Lifex: A freeware for radiomic feature calculation in multimodality imaging to accelerate advances in the characterization of tumor heterogeneity. *Cancer Research* 78, 4786–4789. doi:10.1158/0008-5472.can-18-0125

Nohara, Y., Matsumoto, K., Soejima, H., and Nakashima, N. (2022). Explanation of machine learning models using shapley additive explanation and application for real data in hospital. *Computer Methods and Programs in Biomedicine* 214, 106584

Nuraini, A., Shidik, G. F., Widhiyanti, E. A., Ramadhani, T. O., and Kusuma, E. J. (2025). Performance evaluation of shap and lime for cardiovascular disease prediction using an xai framework. In *2025 International Seminar on Application for Technology of Information and Communication (iSemantic)*. 133–138. doi:10.1109/ISemantic67418.2025.11291855

Osadebey, M., Liu, Q., Fuster-Garcia, E., and Emblem, K. E. (2023). Interpreting deep learning models for glioma survival classification using visualization and textual explanations. *BMC Medical Informatics and Decision Making* 23, 225

Peracchio, L., Nicora, G., Buonocore, T. M., Bellazzi, R., and Parimbelli, E. (2024). *Do You Trust Your Model Explanations? An Analysis of XAI Performance Under Dataset Shift* (Springer Nature Switzerland). 257–266. doi:10.1007/978-3-031-66535-6_28

Rahimi, M. and Rahimi, P. (2023). A short review on the impact of artificial intelligence in diagnosis diseases: Role of radiomics in neuro-oncology: Impact of ai in neuro-oncology diseases: Impact of ai in neuro-oncology diseases. *Galen Medical Journal* 12. doi:10.31661/gmj.v12i.3158

Rahman, A., Hayat, M., Iqbal, N., Alarfaj, F. K., Alkhalaf, S., and Alturise, F. (2025). Enhanced mri brain tumor detection using deep learning in conjunction with explainable ai shap based diverse and multi feature analysis. *Scientific Reports* 15. doi:10.1038/s41598-025-14901-4

Rahman, A., Sohail, S. S., Alam, M. S., Sharma, A., and Mansoor, W. (2024). Detecting brain cancer using explainable ai. In *2024 7th International Conference on Signal Processing and Information Security (ICSPIS)* (IEEE), 1–6

Selvaraju, R. R., Cogswell, M., Das, A., Vedantam, R., Parikh, D., and Batra, D. (2020). Grad-cam: visual explanations from deep networks via gradient-based localization. *International journal of computer vision* 128, 336–359

Tabatabaei, M., Razaei, A., Sarrami, A. H., Saadatpour, Z., Singhal, A., and Sotoudeh, H. (2021). Current status and quality of machine learning-based radiomics studies for glioma grading: a systematic review. *Oncology* 99, 433–443

van Griethuysen, J. J., Fedorov, A., Parmar, C., Hosny, A., Aucoin, N., Narayan, V., et al. (2017). Computational radiomics system to decode the radiographic phenotype. *Cancer Research* 77, e104–e107. doi:10.1158/0008-5472.can-17-0339

Voigtlaender, S., Nelson, T. A., Karschnia, P., Vaios, E. J., Kim, M. M., Lohmann, P., et al. (2025). Value of artificial intelligence in neuro-oncology. *The Lancet Digital Health* 7, 100876. doi:10.1016/j.landig.2025.100876

Zafar, M. R. and Khan, N. (2021). Deterministic local interpretable model-agnostic explanations for stable explainability. *Machine Learning and Knowledge Extraction* 3, 525–541

Zeineldin, R. A., Karar, M. E., Elshaer, Z., Coburger, ·. J., Wirtz, C. R., Burgert, O., et al. (2022). Explainability of deep neural networks for mri analysis of brain tumors. *International journal of computer assisted radiology and surgery* 17, 1673–1683
